# Magnetic Imprinted Polymer-Based Quartz Crystal Microbalance Sensor for Sensitive Label-Free Detection of Methylene Blue in Groundwater

**DOI:** 10.3390/s20195506

**Published:** 2020-09-25

**Authors:** Yufeng Hu, Hanwen Xing, Gang Li, Minghuo Wu

**Affiliations:** School of Ocean Science and Technology, Dalian University of Technology, Panjin 124221, China; m15192562759@163.com (H.X.); lg19161515504@163.com (G.L.); wumh@dlut.edu.cn (M.W.)

**Keywords:** groundwater, magnetic imprinted polymers, methylene blue, quartz crystal microbalance, surfactant

## Abstract

Tiny changes in the mass of the sensor in a quartz crystal microbalance with dissipation monitoring (QCM-D) can be observed. However, the lack of specificity for target species has hindered the use of QCM-D. Here, molecularly imprinted polymers (MIPs) were used to modify a QCM-D sensor to provide specificity. The MIPs were formed in the presence of sodium dodecyl benzene sulfonate. Imprinted layers on Fe_3_O_4_ nanoparticles were formed using pyrrole as the functional monomer and cross-linker and methylene blue (MB) as a template. The MIPs produced were then attached to the surface of a QCM-D sensor. The MIPs-coated QCM-D sensor could recognize MB and gave a linear response in the concentration range 25 to 1.5 × 10^2^ µg/L and a detection limit of 1.4 µg/L. The QCM-D sensor was selective for MB over structural analogs. The MIPs-coated QCM-D sensor was successfully used to detect MB in river water and seawater samples, and the recoveries were good. This is the first time MB has been detected using a QCM-D sensor. Mass is an intrinsic property of matter, so this method could easily be extended to other target species by using different MIPs.

## 1. Introduction

A quartz crystal microbalance with dissipation monitoring (QCM-D) sensor is a simple, very versatile, and sensitive sensor for studying processes that occur on surfaces or within thin films [1]. Mass is a fundamental property of any target analyte, and QCM systems detect analytes by mass, meaning labels are not required [2]. QCM has been used to perform analyses of food, environmental media, and biomedical media [2,3,4]. The complex natures of these sample matrices mean that it is important to develop selective and specific methods for determining target analytes in such matrices. QCM sensors are not selective or specific. This precludes the use of QCM sensors for many applications.

Coating a QCM sensor with molecularly imprinted polymers (MIPs) created by polymerizing functional monomers and cross-linking agents with molecules of the target analyte(s) is an effective way of improving the selectivity of the QCM sensor [5,6,7,8,9]. The two main approaches that have been used to combine MIPs and QCM sensors involve immobilizing pre-prepared MIPs on QCM sensors and in situ polymerization of MIPs on QCM sensors [5]. In situ polymerization gives a homogeneous imprinted layer with a controlled thickness. However, there are some disadvantages to in situ polymerization, including tedious QCM sensor pretreatment, rigorous reaction conditions, and adhesion problems when the imprinted layer is removed. Immobilizing pre-prepared MIPs on QCM sensors avoids these problems. This method allows the characteristics of the MIPs to be retained and the MIPs to be removed easily to give the bare QCM sensors [10]. Magnetic MIPs have been developed to avoid the centrifugation and filtration steps required for conventional MIPs [11]. In addition, magnetic core would increase the binding capacity per unit mass of MIPs through surface molecularly imprinted techniques. Thus, magnetic MIPs was suitable to coat QCM sensors. Polypyrrole (ppy) is a good option for making MIPs because it is non-toxic, environmentally benign, and cheap and simple to prepare [12]. However, ppy MIPs are thin, meaning only small amounts of target will become sorbed to QCM sensor surfaces [13]. It has been found that using a surfactant during the ppy deposition process could increase the amount of ppy that sorbs to the substrate [14].

Dyes are widely used in the leather, paper, pharmaceutical, plastic, and textile industries [15,16]. Emissions of large amounts of dyes in wastewater can negatively affect natural aquatic systems and ecosystems and can affect human health if the dye is transferred through the food chain [17,18]. Some dyes are poorly degradable in the environment and potentially toxic, so have been prohibited from being used in or emitted to areas used for aquaculture in many countries [16]. Methylene blue (MB) is one of the most commonly used dyes. MB has often been used as a model dye for evaluating sorption of nanocomposites. It has been found that ppy-based nanocomposites can efficiently adsorb both cationic and anionic dyes through electrostatic interactions, ion exchange, or π–π interactions [19]. This means that ppy-based MIPs could be used to selectively detect MB.

Here, a QCM-D sensor was combined with MIPs to establish a novel method for detecting MB. First, the MIPs were prepared in the presence of sodium dodecyl benzene sulfonate (SDBS). Pyrrole was used as the functional monomer and cross-linker, and MB was used as the template. The MIPs formed a coating on Fe_3_O_4_ nanoparticles (NPs). Second, QCM-D sensors were coated with the magnetic MIPs using a physical modification method. The detection limit, linear range, and selectivity of the MIPs-coated QCM-D system for MB (the target analyte) were determined. Finally, the system was used to determine MB in river water and seawater to evaluate the applicability of the method to environmental samples.

## 2. Materials and Methods

### 2.1. Chemicals

Dichloromethane, ethanol, FeCl_3_∙6H_2_O, FeSO_4_·7H_2_O, HCl, H_2_O_2_, MB, methanol, rhodamine B (RhB), methyl orange (MO), crystal violet (CV), NaOH, and tetrahydrofuran (THF) were obtained from Tianjin Damao Chemical Reagent Factory (Tianjin, China). Aqueous ammonia (25%) was purchased from Tianjin Kermel Chemical Reagent Co. (Tianjin, China). Pyrrole (J&K Scientific, Beijing, China) was distilled before use. Poly(sodium-*p*-styrene sulfonate) (PSS), polyvinyl chloride (PVC), polyvinyl pyrrolidone (PVP), SDBS, and sodium dodecyl sulfate (SDS) were obtained from Aladdin Industrial Co. (Shanghai, China). All reagents and solvents were of analytical grade. The ultrapure water (18.2 MΩ·cm) used in the experiments was prepared using a Purifier water purification system.

### 2.2. Instrumentation

Topographic images of gold surfaces coated with MIPs were acquired using a Dimension Icon atomic force microscope (Bruker, Santa Barbara, CA, USA) using an air atmosphere and an antimony-doped Si tip (NCHV-A (Bruker), tip radius 8 nm, rectangular cantilever with resonant frequency 320 kHz, spring constant 40 N/m) in tapping mode. Fourier-transform infrared (FT-IR) spectra of the MIPs and non-imprinted polymers (NIPs) were acquired using a Nicolet iN10 MX and iS10 spectrometer (Thermo Fisher Scientific, Waltham, MA, USA) using KBr pellets at room temperature and using 32 cumulative scans and a resolution of 0.09 cm^−1^ in the wavenumber range 500–4000 cm^−1^. Scanning electron microscopy images were acquired using a Nova Nano SEM 450 ultra-high-resolution field emission scanning electron microscope (FEI, Hillsboro, OR, USA). Ultraviolet–visible (UV-Vis) absorption spectra at 664 nm were acquired using a UH-5300 UV-Vis spectrophotometer (Hitachi, Tokyo, Japan). 

### 2.3. Preparation of MIPs

The MIPs were synthesized using an in situ chemical oxidative polymerization technique described in a previous publication with some modifications [13]. Briefly, Fe_3_O_4_ NPs were prepared using a co-precipitation method. The Fe_3_O_4_ NPs were then dispersed in 50.0 mL of a 12% *w*/*w* FeCl_3_ solution and stored until use. An aliquot of the above Fe_3_O_4_ solution was diluted with 100 mL of HCl aqueous solution (pH = 2), then 1.86 g of SDBS was added. After being stirred for 30 min under nitrogen, 10.0 mL of methanol/water (1:1, *v*/*v*) solution containing 0.022 g of MB and 17.3 μL of pyrrole was injected into the above solution and left for 20 min at 0 °C under continuously stirring to form a template–monomer complex. Subsequently, 2.0 mL of HCl aqueous solution (pH = 2) containing 0.45 g of FeCl_3_·6H_2_O was added dropwise. The resulting mixture was allowed to react at 0 °C for 4 h under nitrogen. Afterwards, the product was collected by magnetic separation and then washed with 100.0 mL of aqueous ammonia (pH = 8) and ultrapure water several times to remove template. The obtained MIPs were stored at 4 °C until use. NIPs were prepared in the same way without MB present. MIPs/NIPs-PVP, PSS, or SDS were produced in the same way using PVP, PSS, or SDS as surfactant, respectively, instead of SDBS.

### 2.4. Binding Experiments

The adsorption capacities of the MIPs prepared using different surfactants were determined. Aliquots of the MIPs and NIPs were each mixed with 3 mL of a solution containing 20 mg/L of MB. The mixtures were continually stirred and kept at 25 °C for 1, 5, 10, 15, 30, or 60 min, then the MIPs or NIPs were removed using a magnet. The MB concentration in each supernatant was determined using a UV-Vis spectrophotometer. The amount of MB adsorbed (*Q*) was defined as the difference between the MB concentrations before and after exposure to the MIPs and NIPs and was calculated using the equation

(1) $$Q=\frac{\left(C_{0}-C\right)\times V}{W}$$

where *C*_0_ (mg/L) is the initial MB concentration, *C* (mg/L) is the final MB concentration after the adsorption period, *V* (mL) is the volume of MB solution added, and *W* (g) is the mass of MIPs or NIPs added.

Pseudo-first-order (Equation (2)) and pseudo-second-order (Equation (3)) kinetics equations were used to evaluate the interactions between the MIPs and MB.

(2) $$\ln\left(Q_{e}-Q_{t}\right)=-k_{1}t+lnQ_{e}$$

(3) $$\frac{t}{Q_{t}}=\frac{1}{Q_{e}}t+\frac{1}{k_{2}Q_{e}^{2}}$$

In the equations, *Q_e_* (mg/g) and *Q_t_* (mg/g) are the amounts of MB adsorbed by the MIPs at equilibrium and time t (min), respectively, and *k*_1_ and *k*_2_ are the pseudo-first-order and pseudo-second-order kinetics equation constants, respectively.

### 2.5. Immobilization of MIPs on QCM-D Sensors

An AT-cut quartz crystal chip with a gold electrode (diameter 12 mm, resonance frequency 5 MHz; Renlux Crystal, Shenzhen, China) was used in each QCM-D sensor. Before modification, the gold sensor was cleaned by immersing it in a 5:1:1 *v*/*v*/*v* mixture of ultrapure water, aqueous ammonia, and H_2_O_2_ at 75 °C for 10 min, washing it with water and then ethanol, and then drying it under a stream of nitrogen at room temperature. A 1.5 mg aliquot of the MIPs or NIPs was then dispersed in 1 mL of THF containing 0.5 mg of PVC. A 5 μL aliquot of the mixture was then dropped onto the gold sensor and allowed to dry in air at room temperature. The MIPs- or NIPs-coated sensor was then stored at room temperature until use. 

### 2.6. Preparation of Water Samples

Seawater (from the Daliao River Estuary, N 40.66905°, E 122.11272°) and river water (from a pond on the campus of the Dalian University of Technology, Panjin, China) were analyzed using the MIPs-coated QCM-D sensors to demonstrate that the sensors could be used to detect MB in real environmental samples. Then, 20 mL of seawater or river water was spiked with MB and then passed through a 0.22 μm membrane filter to remove undissolved particles and microorganisms (the pond on the Dalian University of Technology was affected by a cyanobacterial bloom when the sample was collected). Then, the samples were stored at 4 °C until use. 

### 2.7. QCM-D Measurements

A QCM-D system (Q-sense, Gothenburg, Sweden) was used to perform the QCM-D measurements. Each QCM-D measurement was performed at 25 °C (±0.02 °C) using a flow rate of 100 μL/min. The background electrolyte solution was ultrapure water. A background electrolyte solution (ultrapure water) was pumped into the QCM-D module to allow a baseline measurement to be made before a sample was analyzed. The ultrapure water was introduced to the module until the QCM-D signal (ΔF and ΔD) was stable. ΔF and ΔD were then each defined as having values of zero. A solution containing between 1 and 150 μg/L of MB was then injected until the signal became stable. After a certain time, ultrapure water was again pumped into the module to remove unadsorbed MB from the surfaces of the QCM-D sensor. 

QSense Dfind software was used to determine the mass and configuration of the adsorbed layer from the frequency curves (ΔF_i_, i = 3, 5, 7, 9) and dissipation curves (ΔD_i_, i = 3, 5, 7, 9). The Dfind smartfit fitting method was used. 

## 3. Results and Discussion

### 3.1. Preparation and Characterization of the MIPs-Coated QCM-D Sensors

**Scheme 1:**
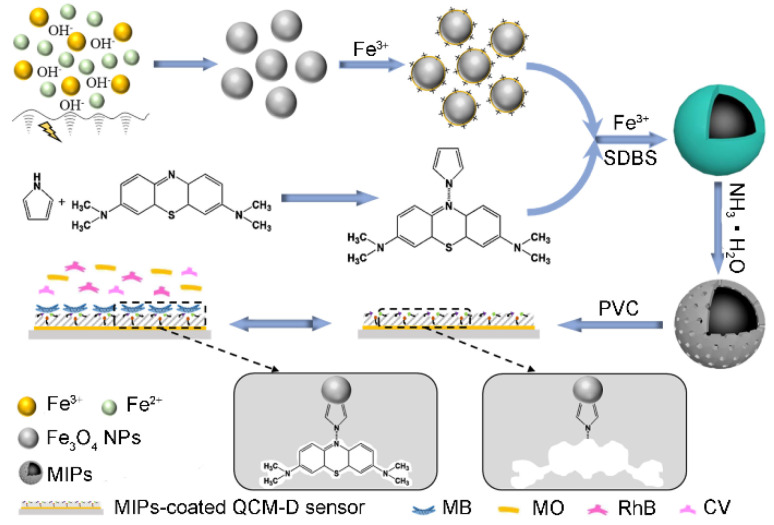
Schematic of the process used to prepare the quartz crystal microbalance with dissipation monitoring (QCM-D) sensors coated with molecularly imprinted polymers (MIPs) (SDBS = sodium dodecyl benzene sulfonate, PVC = polyvinyl chloride, MB = methylene blue, MO = methyl orange, RhB = rhodamine B, CV = crystal violet).

The method used to prepare the MIPs-coated QCM-D sensors is shown in Scheme 1. First, Fe_3_O_4_ NPs were synthesized, then, in the presence of SDBS, MIPs were produced on the Fe_3_O_4_ NP surfaces through an in situ oxidative polymerization technique. The MIPs-coated NPs were then dispersed in THF containing PVC, then coated, with PVC, onto the QCM-D sensor surfaces by evaporating the THF. Finally, the MIPs-coated QCM-D sensors were used to detect MB.

**Figure 1:**
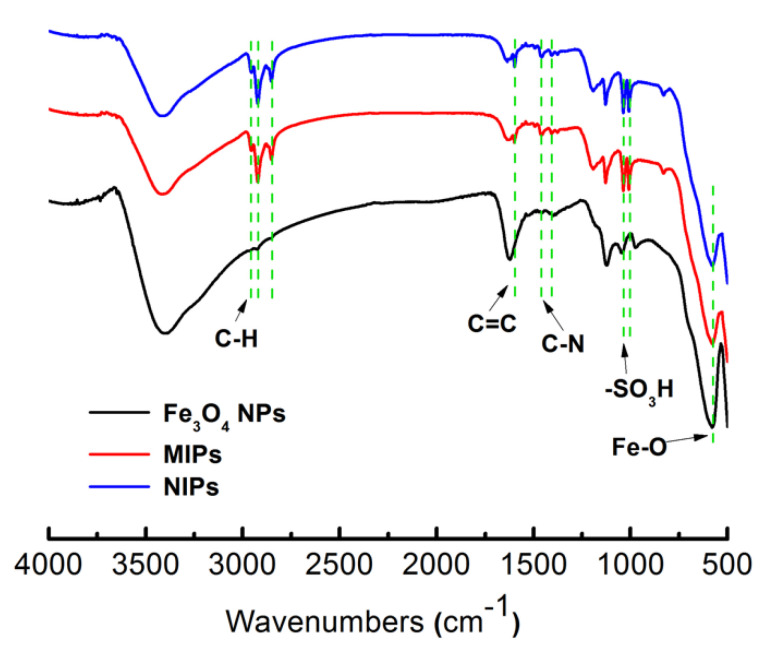
Fourier-transform infrared spectra of the Fe_3_O_4_ nanoparticles (NPs), molecularly imprinted polymers (MIPs), and non-imprinted polymers (NIPs).

The compositions of the materials produced were characterized by acquiring FT-IR spectra of the Fe_3_O_4_ NPs, MIPs, and NIPs particles. The spectra are shown in Figure 1. A peak at 580 cm^−1^ in all three spectra was attributed to Fe–O stretching vibrations. Characteristic ppy peaks at 1544 and 1462 cm^−1^ were found in the MIPs and NIPs spectra but not the pure Fe_3_O_4_ NPs spectrum. The peaks at 1544 and 1462 cm^−1^ were ascribed to stretching of C–C and C–N bonds, respectively, in the pyrrole ring [20,21]. This indicated that a ppy layer had formed on the Fe_3_O_4_ NP surfaces. Peaks at 1008 and 1036 cm^−1^ were attributed to SO_3_H groups [22], which suggested that the MIPs and NIPs contained SDBS molecules. A peak at 2957 cm^−1^ was attributed to stretching vibrations of C–H in SDBS and ppy. The MIPs FT-IR spectrum was almost identical to the NIPs spectrum, indicating that the MB template molecules had been completely removed from the MIPs. These results confirmed that the MIPs and NIPs had been successfully prepared.

**Figure 2:**
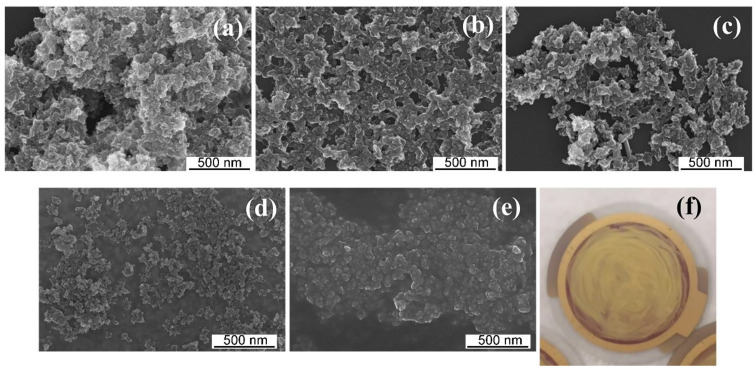
Scanning electron microscopy images of (**a**) the Fe_3_O_4_ nanoparticles, (**b**) the molecularly imprinted polymers (MIPs), (**c**) the non-imprinted polymers (NIPs), (**d**) the MIPs-coated quartz crystal microbalance with dissipation monitoring (QCM-D) sensors, and (**e**) the NIPs-coated QCM-D sensors, and (**f**) a photograph of the MIPs-coated QCM-D sensors.

The Fe_3_O_4_ NPs that were used were synthesized using a co-precipitation method, which is a simple and efficient method for producing superparamagnetic NPs. However, the product easily formed aggregates (Figure 2a). The MIPs encapsulating the Fe_3_O_4_ NPs slightly decreased the tendency of the NPs to form aggregates (Figure 2b,c). The MIPs less readily formed aggregates than did the NIPs. The MIPs and NIPs were coated onto the QCM-D sensor surfaces. The morphologies of the MIPs and NIPs particles did not change even after the MIPs and NIPs were immobilized on the QCM-D sensors (Figure 2d,e). A MIPs or NIPs layer was found on the QCM-D sensor surfaces (Figure 2f).

**Figure 3:**
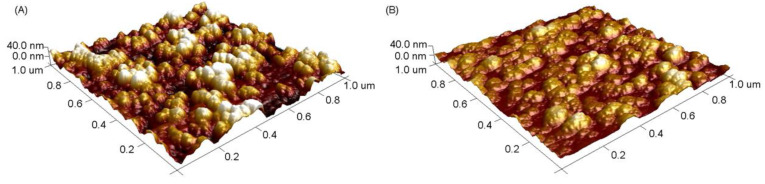
Atomic force microscopy images of quartz crystal microbalance with dissipation monitoring (QCM-D) sensors coated with (**A**) molecularly imprinted polymers (MIPs) and (**B**) non-imprinted polymers (NIPs).

The surface morphologies of MIPs-coated QCM-D sensors were investigated by atomic force microscopy, and the root mean square roughness (Rq) was calculated. The atomic force microscopy images (Figure 3) indicated that an almost homogeneous film had formed on both the MIPs- and NIPs-coated QCM-D sensors. The MIPs-coated QCM-D sensors were rougher (Rq 15.9 nm) than the NIPs-coated QCM-D sensors (Rq 8.9 nm). The MIPs layer was 108.7 nm thick, but the NIPs layer was thinner (61.3 nm thick). The differences between the MIPs- and NIPs-coated QCM-D sensors were attributed to the MIPs and NIPs fabrication processes. The template MB was removed when the MIPs formed, causing the MIPs surfaces to be looser and the MIPs to be larger than the NIPs. The MIPs layer was therefore rougher and thicker than the NIPs layer. These results confirmed that the QCM-D sensor surfaces had been successfully modified with MIPs and NIPs and that the coated QCM-D sensors could be used to recognize certain target analytes.

### 3.2. Selecting the Surfactant Used in the MIPs-Coated QCM-D Sensors

**Figure 4:**
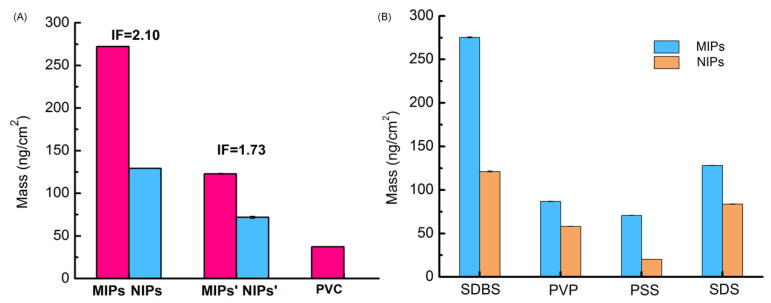
Methylene blue adsorption capacities for the quartz crystal microbalance with dissipation monitoring sensors prepared using different materials. (**A**) Sensors prepared using molecularly imprinted polymers (MIPs) and non-imprinted polymers (NIPs) prepared with and without surfactant and with pure polyvinyl chloride (PVC). (**B**) Sensors prepared using MIPs and NIPs fabricated using various surfactants (sodium dodecyl benzene sulfonate (SDBS), polyvinyl pyrrolidone (PVP), polyvinyl chloride (PVC), poly(sodium-p-styrene sulfonate) (PSS), and sodium dodecyl sulfate (SDS)). IF is the imprinted factor. Mean value ± standard deviation, n = 3.

In a previous study, we found that the ppy-imprinted layer on the Fe_3_O_4_ NPs was very thin, meaning it could adsorb only a small amount of MB [13]. The adsorption capacity of a QCM sensor is crucial to the target being detected because the QCM detection method is based on frequency shifts caused by dramatic changes in the QCM sensor mass. It has previously been found that using an appropriate surfactant can increase the thickness of the coating of the matrix [14]. Therefore, taking the chemical properties of MB and ppy into account, the anionic surfactant SDBS was used to increase the adsorption capacity of the QCM-D sensor. The polymers prepared without SDBS are later called MIPs’ and NIPs’, and the polymers prepared with SDBS are called MIPs and NIPs. As shown in Figure 4A, the changes in mass were markedly stronger for the MIPs- and NIPs-coated QCM-D sensors than the MIPs’- and NIPs’-coated QCM-D sensors, indicating that the presence of SDBS increased the adsorption capacity. The FT-IR spectra indicated that SDBS molecules were present in the MIPs and NIPs. The reason for the adsorption capacity increasing was investigated by calculating the imprinted factor (IF) using the equation IF = Q_MIPs_/Q_NIPs_, where Q_MIPs_ and Q_NIPs_ are the MIPs and NIPs mass shifts, respectively. A higher IF indicated better specificity of the MIPs for the target species [11]. The IF of the MIPs’ was 1.73, but the IF of the MIPs was 2.10, indicating that adding SDBS improved the specificity of the MIPs. The adsorption capacity was increased by interactions between SDBS and the target analyte and because the SDBS increased the thickness of the ppy-imprinted layer, which allowed many more imprinted cavities to occur in the MIPs and made the system more specific to the target analyte. These results indicated that the presence of SDBS improved the adsorption capacities of the MIPs and NIPs. The MIPs were coated onto the QCM-D sensor surfaces with PVC, so the effect of PVC on adsorption of the target analytes was also evaluated. The adsorption capacities of the MIPs and NIPs were compared, and little MB was found to be adsorbed by the PVC-coated QCM-D sensor.

The abilities of MIPs-coated QCM-D sensors prepared using different anionic surfactants (PSS or SDS) and a non-ionic surfactant (PVP) to adsorb MB were tested, and the results are shown in Figure 4B. MIPs and NIPs prepared using SDBS as a surfactant adsorbed more MB than did the MIPs and NIPs-PVP, PSS, or SDS, indicating that SDBS was the most appropriate surfactant for preparing the MIPs and NIPs. Interestingly, the adsorption capacity of the MIPs-PSS was lower than the adsorption capacity of the MIPs-PVP. This was because the MIPs-PVP could not completely cover the QCM-D sensor surfaces at the MIPs concentration that was used. The differences between the MIPs and NIPs prepared using the different surfactants were therefore investigated by performing kinetics tests using all of the types of MIPs and NIPs that were produced.

**Figure 5:**
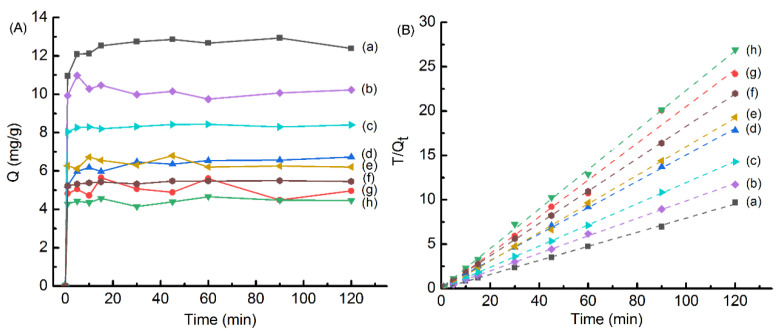
(**A**) Adsorption kinetics curves and (**B**) pseudo-second-order fitting curves for the molecular imprinted polymers (MIPs) prepared using different surfactants. (a) MIPs, (b) MIPs-PSS, (c) MIPs-SDS, (d) MIPs-PVP, (e) non-imprinted polymers (NIPs)-PSS, (f) NIPs-SDS, (g) NIPs, and (h) NIPs-PVP. PSS = poly(sodium-p-styrene sulfonate), SDS = sodium dodecyl sulfate, PVP = polyvinyl pyrrolidone.

As shown in Figure 5A, almost all of the MIPs and NIPs reached adsorption equilibrium in <5 min, indicating that the ppy-based MIPs and NIPs had strong abilities to sorb MB and that pyrrole was a suitable functional monomer. All of the MIPs adsorbed larger amounts of MB than did the NIPs, indicating the importance of the effective imprinted cavities in the MIPs. The *Q_e_* values decreased in the order MIPs > MIPs-PSS > MIPs-SDS > MIPs-PVP, indicating that the MIPs prepared using SDBS gave the highest MB adsorption capacity. There were two reasons for this. First, SDBS, being an anionic surfactant, could interact with MB through electrostatic and π–π interactions. Second, the presence of SDBS increased the thickness of the imprinted ppy layer, which caused more imprinted cavities to form. The polyelectrolyte PSS would have increased the negative charge density and hydrophilicity of the MIPs that were produced. Unlike SDBS and PSS, SDS would only have interacted with MB through electrostatic interactions (not through π–π interactions). This explained why the adsorption capacities were lower for the MIPs-PSS and SDS than for the MIPs prepared using SDBS. The non-ionic surfactant PVP would not have interacted with MB through electrostatic interactions. This explained why the MIPs-PVP had the lowest MB adsorption capacity. 

Interactions between the MIPs and MB were investigated further using the pseudo-first-order and pseudo-second-order kinetics equations. As shown in Table 1, the correlation coefficients (R^2^) for the pseudo-first-order kinetics equation were <0.5 but the R^2^ values for the pseudo-second-order kinetics equation were >0.99. The *Q_eq_* values calculated using the pseudo-first-order kinetics equation were very similar to the experimental values. The MB adsorption kinetics results (Figure 5B) for the MIPs and NIPs were therefore best described using the pseudo-second-order kinetics equation, indicating that chemical adsorption played a crucial role in adsorption of MB onto the MIPs and NIPs. These results indicated that SDBS was the best surfactant for preparing the MIPs.

### 3.3. Analytical Performance of the MIPs-Coated QCM-D Sensor

**Figure 6:**
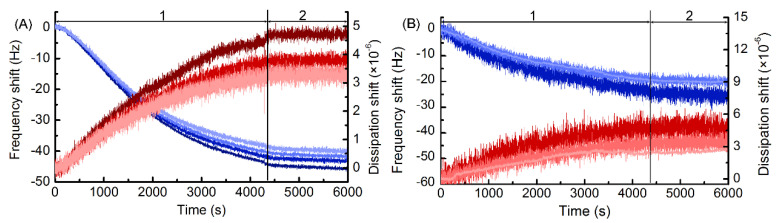
Frequency and dissipation shifts, in response to methylene blue attachment to (**A**) the quartz crystal microbalance with dissipation monitoring sensor coated with the molecularly imprinted polymer and (**B**) the quartz crystal microbalance with dissipation monitoring sensor coated with the non-imprinted polymer. 1 indicates the introduction of methylene blue and 2 indicates rinsing with water.

A typical QCM-D sensor experiment had three stages, (a) introduction of the buffer, (b) adsorption of MB, and (c) washing off non-bound target analyte molecules. Introducing the buffer would cause the frequency shift to remain stable when the solution containing MB was pumped into the QCM-D system. Adsorption of MB by the QCM-D sensor would cause the frequency to change markedly. As shown in Figure 6, the frequency shifted more for the MIPs-coated QCM-D sensor than the NIPs-coated QCM-D sensor, indicating that the MIPs-coated QCM-D sensor more readily bound MB because of the presence of imprinted cavities. The marked decrease in the frequency caused a slight increase in dissipation from both the MIPs-coated and NIPs-coated QCM-D sensor systems. It has previously been found that changes in dissipation in QCM-D sensors correlate with changes in the viscoelastic characteristics of the sensor [23]. The similar changes in dissipation in the MIPs-coated and NIPs-coated QCM-D sensors therefore indicated that both sensors had comparable coatings. Once the frequency became stable, ultrapure water was pumped into the sensor to remove any unbound MB. No clear changes in frequency or dissipation occurred (Figure 6), indicating that minimal mass was lost, and minimal structural changes occurred in the sensor surfaces. This indicated that the MIPs-coated QCM-D sensor could be used to detect MB.

**Figure 7:**
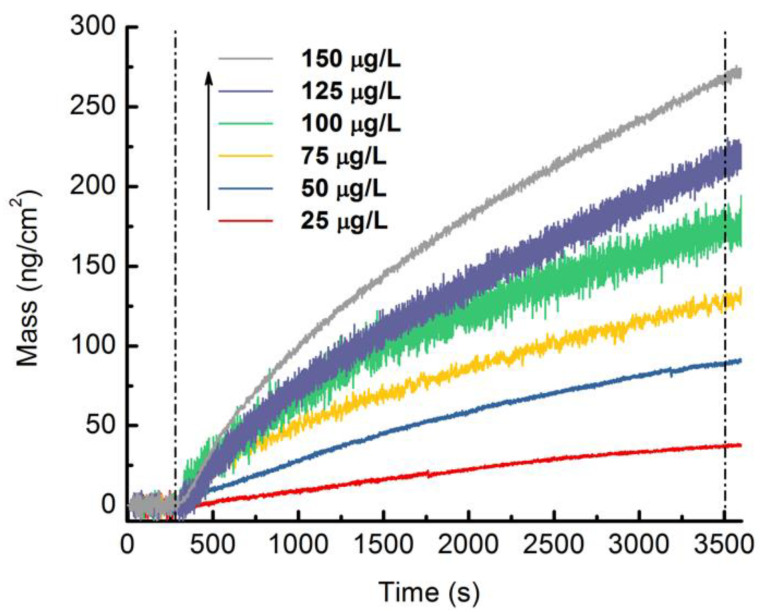
Changes in mass of the quartz crystal microbalance with dissipation monitoring sensor coated with molecular imprinted polymer at different methylene blue concentrations.

The QCM-D was used to study the surface-bound MB further to allow the surface concentration of the MB to be quantified. Like in the tests described above, introducing MB caused the MIPs-coated QCM-D sensor frequency to change markedly because of the mass of MB that adsorbed onto the sensor surfaces. The change in mass of the sensor was calculated using QSense Dfind software. The change in mass of the MIPs-coated QCM-D sensor increased gradually as the MB concentration increased (Figure 7). The response was linear in the MB concentration range 25 to 1.5 × 10^2^ µg/L (*Y* = 1.8809*x* − 9.4638, R^2^ = 0.9907). The limit of detection (the concentration giving a signal-to-noise ratio of 3) was 1.4 µg/L. We concluded that the MIP-coated QCM-D sensor could be used to detect MB.

### 3.4. Selectivity and Reproducibility of the MIPs-Coated QCM-D Sensor

**Figure 8:**
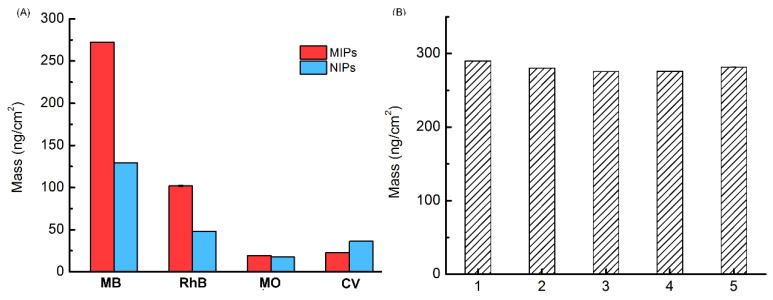
(**A**) Changes in mass of the quartz crystal microbalance with dissipation monitoring sensors coated with molecular imprinted polymers (MIPs) and non-imprinted polymers (NIPs) in the presence of methylene blue (MB), rhodamine B (RhB), methyl orange (MO), and crystal violet (CV). (**B**) Reproducibility of the change in mass of the quartz crystal microbalance with dissipation monitoring sensor coated with MIPs in response to the presence of 150 µg/L of MB. Mean value ± standard deviation, n = 3.

Selectivity is a crucial factor for a sensor. The selectivity of the MIPs-coated QCM-D sensor was evaluated by performing competitive binding experiments using analogs of MB (RhB, MO, and CV). RhB and CV are non-azo dyes, like MB, and MO is an azo dye. The changes in mass of the MIPs- and NIPs-coated QCM-D sensors in the presence of the four-target species were determined, and the results are shown in Figure 8A. The largest change in mass was for MB. This would have been because of the imprinted cavities formed during the fabrication of the MIPs. The MB fitted the templates and therefore readily became bound to the MIPs and caused large changes in the mass of the MIPs-coated QCM-D sensor. The mass of the QCM-D sensor changed little in the presence of MO, possibly because of the different functional groups and molecular structures of MB and MO. Moreover, QCM-D is a device that monitoring mass variation of its sensor, the molecular weight of the target adsorbed onto the sensor surface should be considered. The molecular weights of MB, RhB, MO, and CV were 319.86, 479.01, 327.33, and 407.98 g/mol, respectively. Even though the MB possessed lowest molecular weight, it caused the highest mass change of the QCM-D sensor, further proving that there were much more MB molecules adsorbed onto the MIPs-coated QCM-D sensor surface. These results indicated that the MIPs-coated QCM-D sensor was very selective and specific, which would favor the practical use of the sensor to analyze real samples. 

The reproducibility of the MIPs-coated QCM-D sensor is an important advantage over other methods. Physically modified QCM-D sensors generally give more unstable results than chemically modified QCM-D sensors. We found that MIPs-coated QCM-D sensors prepared in different batches gave similar changes in mass in the presence of MB (Figure 8B), indicating that the method had a good degree of reproducibility and the physical modification method could be used to fabricate QCM-D sensors for detecting MB. Moreover, the standard deviations of triplicates in each run were below 1.0, suggesting an excellent stability of the established method.

### 3.5. Application of the MIPs-Coated QCM-D Sensor

The practical use of the established method was assessed by analyzing river water and seawater samples. MB was not detected in any of the samples by high-performance liquid chromatography. A recovery experiment was therefore performed. The matrix calibration curve of each sample was built by spiking the blank sample with 25 to 1.5 × 10^2^ µg/L MB solution. The matrix effect was defined as *α_matrix_*/*α_solvent_* (where *α_matrix_* and *α_solvent_* were the slope of matrix calibration curve and ultrapure water calibration curve, respectively), and the results were listed in Table 2. The weak matrix effects for river water and seawater indicated a good accuracy of the established detection method. Moreover, the samples were spiked with MB at three concentrations (50, 100, and 150 µg/L). As shown in Table 3, the MB recoveries for river water were 93.6%–96.8% and the relative standard deviations were 4.1%–4.2% and the MB recoveries for seawater were 86.3%–93.3% and the relative standard deviations were <2.8%. These results indicated that the method offers promise for accurately determining MB in groundwater.

The QCM-D sensor, surface plasmon resonance, and surface enhanced Raman scattering rely on the surface chemistry of the sensors to sensitively detect the target analyte [22]. The good sensitivities, rapid responses, and lack of need for label mean that these methods can be used to detect target analytes at low concentrations. The limit of detection for MB was lower for the QCM-D sensor than for surface plasmon resonance and surface enhanced Raman scattering (Table 4), suggesting that the QCM-D sensor could be used to determine MB at low concentrations. Other common methods (capillary electrophoresis, fluorescence spectroscopy, high-performance liquid chromatography, and spectrophotometry) have been used to determine MB. The QCM-D sensor gave comparable limits of detection and linear ranges to these methods.

## 4. Conclusions

The magnetic MIPs were coated onto the surface of a QCM-D sensor to fabricate a sensor. To the best of our knowledge, this is the first time magnetic MIPs and QCM-D have been combined to detect a dye. The method was found to be simple and efficient, but the MIP layer was somewhat thick and non-uniform, which would negatively affect the sensitivity and cause a long adsorption equilibrium-attainment time of the QCM-D sensor. A spin-coating procedure may be better to produce a thinner coating. And in order to shorten the detection time, it is possible to choose a time when the mass of the developed QCM-D sensor begins to change significantly rather than waiting for adsorption equilibrium.

## Figures and Tables

**Table 1:** Kinetics parameters for methylene blue adsorption by the molecularly imprinted polymers (MIPs) and non-imprinted polymers (NIPs) prepared using different surfactants (sodium dodecyl benzene sulfonate (SDBS), sodium dodecyl sulfate (SDS), poly(sodium-p-styrene sulfonate) (PSS), and polyvinyl pyrrolidone (PVP)).

Materials	*Q_e_* (mg·g^−1^)	Pseudo-Second-Order Model		
		*k*_2_ (g·mg^−1^·min^−1^)	*Q_eq_* (mg·g^−1^)	R^2^
MIPs	12.9	0.169	12.9	0.9998
NIPs	5.6	0.254	5.4	0.9904
MIPs-SDS	8.4	1.157	8.4	0.9999
NIPs-SDS	5.5	0.974	5.5	0.9999
MIPs-PSS	11.0	0.894	10.1	0.9994
NIPs-PSS	6.8	0.401	6.7	0.9975
MIPs-PVP	6.7	0.123	6.7	0.9995
NIPs-PVP	4.5	0.796	4.5	0.9991

Notes: *Q_e_* was determined from the experimental data, *Q_eq_* was calculated from the pseudo-second-order kinetics equation.

**Table 2:** The matrix calibration curve and matrix effect of river water and seawater.

Samples	Matrix Calibration Curve	Matrix Effect
River water	y=1.6157x + 63.473	115%
Seawater	y=1.5590x + 48.788	128%

**Table 3:** Recoveries of methylene blue in different samples (n = 3).

Samples	C_added_ (µg/L)	C_found_ (µg/L)	Recovery (%)	RSD (%, n = 3)
River water	50	48.4	96.8	4.2
	100	95.6	96.4	4.1
	150	140.4	93.6	4.1
Seawater	50	46.6	93.3	0.9
	100	92.5	92.5	2.8
	150	129.5	86.3	1.3

**Table 4:** Comparison of the method with other methods for detecting methylene blue.

Methods	Linear Range (µg/L)	LOD (µg/L)	Reference
CE	1.0 × 10^3^–1.2 × 10^6^	1.0 × 10^3^	[24]
UV-Vis spectrophotometry	2.0–6.0 × 10^2^	0.6	[15]
HPLC-UV/Vis	1.6–6.0 × 10^2^	0.5	[15]
Fluorescence	16–3.0 × 10^3^	3.2	[25]
SERS	3.2–3.2 × 10^8^	3.2	[26]
MIPs-SERS	-	3.7 × 10^2^	[27]
SPR	1.6 × 10^3^–1.6 × 10^5^	1.6 × 10^3^	[28]
QCM-D	25–1.5 × 10^2^	1.4	This study

Note: SERS = surface enhanced Raman scattering, CE = capillary electrophoresis, SPR = surface plasmon resonance, UV/Vis = ultraviolet–visible absorption spectrometry.
